# Insulin secretion hot spots in pancreatic β cells as secreting adhesions

**DOI:** 10.3389/fcell.2023.1211482

**Published:** 2023-05-26

**Authors:** Margret A. Fye, Irina Kaverina

**Affiliations:** Kaverina Lab, Department of Cell and Developmental Biology, Vanderbilt University, Nashville, TN, United States

**Keywords:** glucose-stimulated insulin secretion, pancreatic β cell, focal adhesion, microtubule, actin cytoskeleton, mechanosensitive

## Abstract

Pancreatic β cell secretion of insulin is crucial to the maintenance of glucose homeostasis and prevention of diseases related to glucose regulation, including diabetes. Pancreatic β cells accomplish efficient insulin secretion by clustering secretion events at the cell membrane facing the vasculature. Regions at the cell periphery characterized by clustered secretion are currently termed insulin secretion hot spots. Several proteins, many associated with the microtubule and actin cytoskeletons, are known to localize to and serve specific functions at hot spots. Among these proteins are the scaffolding protein ELKS, the membrane-associated proteins LL5β and liprins, the focal adhesion-associated protein KANK1, and other factors typically associated with the presynaptic active zone in neurons. These hot spot proteins have been shown to contribute to insulin secretion, but many questions remain regarding their organization and dynamics at hot spots. Current studies suggest microtubule- and F-actin are involved in regulation of hot spot proteins and their function in secretion. The hot spot protein association with the cytoskeleton networks also suggests a potential role for mechanical regulation of these proteins and hot spots in general. This perspective summarizes the existing knowledge of known hot spot proteins, their cytoskeletal-mediated regulation, and discuss questions remaining regarding mechanical regulation of pancreatic beta cell hot spots.

## Introduction

Pancreatic β cells are responsible for secreting insulin into the bloodstream in response to glucose stimulation, in order to promote glucose uptake in tissues. The direction and levels of secretion must be optimized, to ensure that insulin gets into the vasculature and that the right amount of insulin is secreted. To accomplish this, β cells create subcellular structures known as hot spots, which promote clustered and targeted secretion to the vasculature (Low et al., 2014; Gan et al., 2017; Almaça et al., 2018; Ohara-Imaizumi et al., 2019; Trogden et al., 2020). These hot spots contain scaffolding proteins common to the neuronal presynaptic active zone such as ELKS and liprin (Low et al., 2014; Gan et al., 2017), and proteins associated with the cytoskeletal and adhesion machinery such as integrin β1 (Gan et al., 2018), KANK1, and LL5β (Noordstra et al., 2022). Based on their molecular composition and known regulation, the hot spot structure may be more precisely termed a “secreting adhesion.”

The microtubule (MT) and actin cytoskeletons are each known to regulate insulin secretion (Thurmond et al., 2003; Jewell et al., 2008; Zhu et al., 2015; Bracey et al., 2020; Ho et al., 2020), and MTs were recently found to negatively regulate clustered insulin secretion at hot spots, specifically (Trogden et al., 2020). Despite this, the subcellular organization, dynamics, and polarity of the cytoskeleton remains uncharacterized at hot spots. In addition, the function of the cytoskeleton at hot spots is largely undetermined. Specifically, the mechanisms by which the cytoskeleton regulates clustered secretion is unknown. Understanding the cytoskeleton structure at hot spots would provide further information as to the mechanism underlying hot spot assembly and function.

## Characteristic hot spot components and their functions

### Integrins and focal adhesions

The effects of the extracellular matrix (ECM) on β cell identity and function have been well-studied, and ECM-cell signaling, particularly via integrins, is necessary for β cell differentiation, glucose sensitivity, and insulin secretion (Parnaud et al., 2006; Krishnamurthy et al., 2008; Rondas et al., 2011; Diaferia et al., 2013). Integrins are heterodimeric transmembrane proteins with an α and β subunit, serving as the link between ECM proteins and focal adhesion proteins in the cytoplasm. Laminin5, an ECM protein associated with the vasculature, has been shown to be specifically necessary to β cell spreading and insulin secretion through its binding of integrin β1 (Parnaud et al., 2006). Integrin β1 plays a crucial role in maintaining normal glucose-stimulated insulin secretion [GSIS (Bosco et al., 2000; Kaido et al., 2004; Krishnamurthy et al., 2008; Riopel et al., 2011; Rondas et al., 2012; Diaferia et al., 2013; Peart et al., 2017; Kakinuma et al., 2008)], and has become a strong candidate for defining β cell polarity (Gan et al., 2018; Jevon et al., 2022), owing to the signaling it facilitates between the islet capillaries and β cells (Spelios et al., 2015). Integrin activation leads to FAK activation, which is important for not just insulin granule (IG) docking and insulin secretion (Rondas et al., 2011; Cai et al., 2012; Rondas et al., 2012; Arous et al., 2013; Jevon et al., 2022), but, specifically, positioning of key scaffolding protein ELKS and site-specific Ca2+ influx (Ohara-Imaizumi et al., 2019; Jevon et al., 2022), contributing to vascular-oriented insulin secretion (Gan et al., 2018). This integrin activity at the vascular face of the β cell, and FAK activation downstream, imply a central role for focal adhesion signaling in establishing the site of hot spot secretion. Moreover, loss of adhesion-based structures, such as during islet isolation, can lead to a loss of β cell identity (Negi et al., 2012), indicating that adhesion signaling is necessary for β cell identity and function.

### KANK1

KANK1, named for its ankyrin repeats, is a mechanosensitive protein typically localized to the outer edges of focal adhesions (Sun et al., 2016), where it binds to talin (Bouchet et al., 2016), another mechanosensitive focal adhesion protein which connects F-actin to integrins and therefore the ECM. KANK1 interacts with liprin-β1 and LL5β at regions strongly considered to be hot spots (van der Vaart et al., 2013; Bouchet et al., 2016). Thus, KANK1 connects adhesions with secretory machinery (focal adhesions and liprins) and acts as a “seed” for cortical MT stabilizing complexes (CMSCs; which include LL5β), bringing together multiple components of putative hot spot structures (Bouchet et al., 2016). Additionally, since KANK proteins play a role in inhibition of the small GTPase Rac1 and actin polymerization (Chandra Roy et al., 2009), they could contribute to actin cytoskeleton remodeling at hot spot sites during secretion.

### Liprins

Liprin-α1 and liprin-β1 are two scaffolding proteins known to localize to presynaptic active zones, a neuronal structure similar to hot spots, and are therefore suspected to localize to β cell hot spots. Supporting this is the finding that liprin is enriched at the vascular face of β cells (Low et al., 2014; Gan et al., 2017; Cottle et al., 2021). Liprin-α1was originally characterized for its role in interacting with LAR tyrosine phosphatases, through which it can promote focal adhesion disassembly, thus altering cell-matrix interactions (Serra-Pages et al., 1995). Liprins have been established as part of the presynaptic active zone and have many overlapping scaffolding functions with ELKS proteins. They are known to interact with and form complexes with ELKS, LL5β, and KANK1, as part of the CMSC (Lansbergen et al., 2006; van der Vaart et al., 2013; Bouchet et al., 2016). In particular, liprin-β1’s interaction with KANK1 serves as a physical linkage between focal adhesions (via talin) and CMSCs (van der Vaart et al., 2013; Bouchet et al., 2016). Positioning of liprins and other hot spot proteins including ELKS requires integrin and subsequent focal adhesion activation (Jevon et al., 2022). Despite the known localization of liprins and their thorough characterization as adhesion proteins, the role of liprins in clustered insulin secretion at hot spots is still largely uncharacterized.

### LL5β

LL5β is a membrane-associated protein which gains its membrane localization upon activation of PI3K and subsequent phosphorylation of PIP2 to PIP3 (Paranavitane et al., 2003; Kishi et al., 2005). LL5β then binds PIP3 to associate with the membrane. LL5β is also known to bind gamma-filamin, an actin crosslinking protein (Paranavitane et al., 2003) and to localize adjacent to focal adhesions (Lansbergen et al., 2006; Noordstra et al., 2022). LL5β association with active zones and hot spots is established through its known interactions with ELKS (Lansbergen et al., 2006) and liprins (van der Vaart et al., 2013), and forms overlapping/co-localizing structures with ELKS patches, forming its own LL5β patches. LL5β is also known to interact with CLASP2 (Lansbergen et al., 2006; Basu et al., 2015), a plus-end MT-binding protein, and is thus associated with the plus ends of MTs and serves as a mechanism to attach MT plus ends to the cell cortex (Hotta et al., 2010). LL5β knockdown resulted in a decrease of cortical MTs (Lansbergen et al., 2006). At the neuromuscular junction, LL5β is required for MT targeting via CLASP2 and cooperative MT- and actin-mediated acetylcholine receptor targeting (Basu et al., 2015). Importantly, LL5β is required for IG clustering and hot spot function (Noordstra et al., 2022), implicating MT targeting and therefore clustering of exocytotic machinery in the downstream pathway of LL5β.

### ELKS

ELKS is a scaffolding protein, so named for its repeats of glutamate (E), leucine (L), lysine (K), and serine (S). ELKS is best known as a part of the cytomatrix at the presynaptic active zone (CAZ) of neurons (Monier et al., 2002; Ohtsuka et al., 2002; Wang et al., 2002). At CAZ, this scaffolding protein participates in physical separation of presynaptic vesicle capture and exocytosis (Nyitrai et al., 2020). As one of many similarities between the exocytic processes in neurons and pancreatic β cells, ELKS localizes and clusters in β cells around the vasculature (Ohara-Imaizumi et al., 2005; Low et al., 2014) and in regions of dense IG exocytosis (Ohara-Imaizumi et al., 2005). ELKS’ scaffolding function allows physical tethering of many active zone/hot spot proteins, including RIM2, liprinα1, piccolo/bassoon, MICAL3 (and Rab8a), Rab6, and others (Ohara-Imaizumi et al., 2005; Grigoriev et al., 2011, and for review see Sudhof, 2012). This scaffolding function and co-localization with secretion hot spots implicates ELKS as the primary component of pancreatic β cell hot spots.

Indeed, β cell-specific ELKS knockout in mice results in impaired GSIS and glucose tolerance, and impaired β cell Ca2+ flux (Ohara-Imaizumi et al., 2019). ELKS interacts with L-type voltage-dependent calcium channels (VDCCs) as part of the Ca2+ flux machinery tied to SNARE exocytosis machinery and could also alter the current of VDCCs to influence local exocytosis event probability (Ohara-Imaizumi et al., 2019). ELKS has also been shown to facilitate secretion specifically at focal adhesions in non-β cell models of exocytosis (Fourriere et al., 2019), suggesting a potential interaction with focal adhesion machinery in β cells as well. Thus, in recent years, ELKS has served as the gold standard for marking pancreatic β cell hot spots.

### Other hot spot proteins

Importantly, here we have not described some proteins which are essential for secretion and are present but are not concentrated at hot spots and thus are unlikely to direct targeted secretion. These include ion channels, SNAREs, etc., (Low et al., 2014; Ohara-Imaizumi et al., 2019). A good example of such molecules are VDCCs, which are distributed all over the plasma membrane yet get specifically activated at hot spots (Ohara-Imaizumi et al., 2019; Jevon et al., 2022). In addition, some likely specific hot spot proteins have been less characterized with regards to clustered insulin secretion. These include Munc18c, a critical component of exocytotic machinery, which has also been found to impair GSIS when depleted in mouse islets (Oh & Thurmond, 2009). RIM2a is another putative hot spot protein, known to bind Rab3A on IGs and play a role in the docking step of IG exocytosis (Yasuda et al., 2010). These proteins and more likely also contribute to hot spot formation and clustered insulin secretion.

## Role of the cytoskeleton in hot spot organization and clustered secretion

### Microtubules

Though MTs have been known for years to play a role in insulin secretion, the field’s understanding of this role has evolved over time. Originally, the role of MTs was thought to be restricted to transporting IGs from the Golgi to the membrane via kinesin-1 motors (Boyd et al., 1982; Varadi et al., 2003). While new IGs are indeed distributed by non-directional IG transport all over the cell (Tabei et al., 2013; Hoboth et al., 2015; Zhu et al., 2015), it has since been shown that in functional β cells, which already have multiple preexisting IGs in their cytoplasm, MTs play a stronger role in restricting, rather than promoting, insulin secretion response (Zhu et al., 2015; Bracey et al., 2020; Ho et al., 2020). Several lines of evidence show this: nocodazole treatment, which depolymerizes MTs, leads to enhanced insulin secretion (Zhu et al., 2015), and MTs are destabilized under high glucose, leading to enhanced insulin secretion (Zhu et al., 2015; Ho et al., 2020; Trogden et al., 2020). Structural studies have also shown that IGs cluster at the membrane near MTs (Muller 2020), and that in the absence of MTs, there is enhanced accumulation of IGs at the membrane (Bracey et al., 2020; Hu et al., 2021). This suggests that MTs act to reduce the number of readily releasable IGs at the secretion sites. This function is attributed specifically to a MT subset which is oriented laterally to the plasma membrane (Bracey et al., 2020; Ho et al., 2020). Rather than acting as a stationary barrier, however, these submembrane MTs have been shown to actively transport IGs away from the membrane, suggesting that this activity supports withdrawal of excessive IGs (Zhu et al., 2015; Bracey et al., 2020; Ho et al., 2020). Interestingly, MT destabilization specifically enhances clustered insulin secretion, including more hot spots per cell and more secretions per hot spot (Trogden et al., 2020). Despite this evidence, however, it is unknown through what mechanism MTs help to regulate clustered secretion at hot spots.

Though we do not know precisely what the role of MTs at the hot spot is, association with some hot spot and cortical proteins supports its association with such structures. The ability of LL5β to bind CLASP2 anchors MT plus ends to the cell cortex (Basu et al., 2015; Bouchet et al., 2016) and these complexes are known to play a role in exocytosis (for review see Noordstra and Akhmanova, 2017). It is also known that KANK1 recruits and binds Kif21A, a kinesin-4 which regulates MT length at the cortex (van der Vaart et al., 2013). These findings, along with the finding that MT depolymerization promotes clustered secretion (Trogden et al., 2020), suggest there is in fact a distinct role for MTs at the hot spot.

### Actin

A variety of roles have been proposed for the actin cytoskeleton in GSIS regulation. It has been well-established that the cortical actin cytoskeleton undergoes remodeling upon glucose stimulation (Nevins and Thurmond, 2003; Naumann et al., 2017). It has been proposed that F-actin may play a negative regulatory role in GSIS by binding to SNARE machinery (Thurmond et al., 2003), namely, syntaxin-4 and controlling access of IGs to the exocytotic machinery (Jewell et al., 2008). In agreement with this, a simple physical barrier function was proposed for cortical actin (Wang et al., 1990; Heaslip et al., 2014), where actin serves as the primary cytoskeleton regulator of IGs under basal conditions, but MT-dependent delivery take over upon glucose stimulation. Actin-dependent restriction of secretion likely applies predominantly to older IGs, which associate with F-actin and are less likely to be secreted (Hoboth et al., 2015).

In contrast, some studies indicate that actin may have a transport role delivering IGs to the membrane on a short-distance scale via myosin II (Arous et al., 2013), or non-conventional myosins myo1b (Tokuo et al., 2021) and myosin 5a (Ivarsson et al., 2005; Varadi et al., 2005).

However, specific actin dynamics and function at secretion hot spots have not been sufficiently studied. It is interesting in the context of this review that actomyosin contractility, a major regulator of cell adhesions, is also important for GSIS regulation. Specifically, myosin IIA is also required for glucose-induced actin remodeling and focal adhesion assembly to promote insulin secretion (Arous et al., 2013), and pharmacological myosin II inhibition resulted in a decrease in intracellular IG clustering and clustered secretion (Noordstra et al., 2022).

## Interplay of cytoskeletal and adhesion machinery at insulin secretion hot spots

Components of different cytoskeletal systems which are found at insulin secretion sites indicate a potential for regulatory crosstalk between adhesion proteins, actin cytoskeleton, and MTs. On one hand, integrin-dependent signaling, via FAK activation, likely initiates the assembly of hot spot machinery, including adhesion and actin components as described above. Consequently, assembly of adhesion plaques can promote formation of the CMSC, via talin-KANK-LL5β interaction (van der Vaart et al., 2013; Bouchet et al., 2016) and LL5β binding to CLASP2 (Lansbergen et al., 2006) and/or KIF21A (van der Vaart et al., 2013). While these MT-capturing mechanisms have been described in other cell types, they could potentially enhance MT anchoring and MT-dependent IG transport at secreting adhesions in β cells as well. Reciprocal regulation of focal adhesions by MTs (Kaverina et al., 1998; 1999) is also well-described in other cell types. Interestingly, MT-dependent adhesion dynamics are tuned by FAK activity (Ezratty et al., 2005), which, as indicated above, is critical for secretion at hot spots, suggesting a potential bi-directional interplay of regulatory pathways at this location.

Interestingly, adhesion machinery is tightly mechanically regulated. Some critical components of cortical hot spot machinery are known mechanosensors, such as talin and KANK1. KANK proteins directly bind the rod domain of talin and couple integrin activation to actomyosin contractility (Sun et al., 2016), which has recently been shown to be essential for supporting clustered secretion (Noordstra et al., 2022). This suggests that previously described dependency of GSIS on the mechanical properties on the islet microenvironment (Parnaud et al., 2006; Nyitray et al., 2014; Geron et al., 2015; Zhang et al., 2021) likely acts in a β cell locally, at the “secreting adhesion” sites.

It is plausible to suggest that both contractility and stiffness of the microenvironment would lead to the growth/stabilization of the secretion hot spot, similar to mechanical facilitation of adhesion assembly (Dumbauld et al., 2010; Rafiq et al., 2019). Subsequently, such regulation would promote cortical MT capture at these sites. In turn, MTs could potentially participate in regulation of cell tension and contractility: MT depolymerization is known to release a RhoA GEF-H1, which facilitates myosin II contractility via the RhoA pathway (Krendel et al., 2002; Chang et al., 2008; Heck et al., 2012; Seetharaman et al., 2021).

Existing evidence from β cells, combined with findings from other cell systems, indicates that hot spots of secretion at the vascular face of β cells involve molecular machinery associated with several cytoskeletal systems, which exist in constant regulated cross-talk.

## Conclusion

**FIGURE 1:**
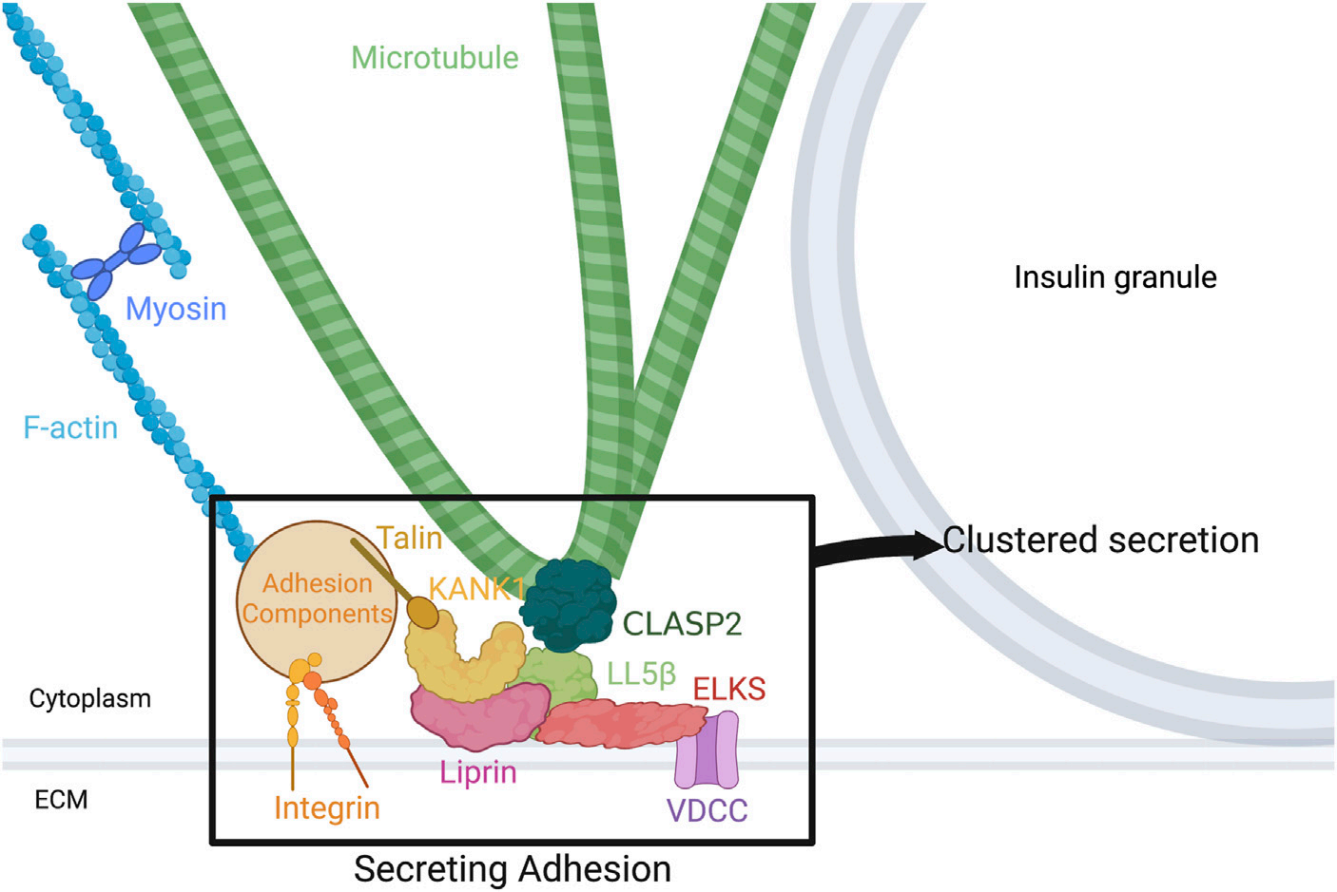
Components of the secreting adhesion and their relation to the cytoskeleton. From the outside-in, integrins link the ECM to focal adhesions and F-actin. Talin, one focal adhesion protein, binds KANK1, tying the focal adhesion to CMSC and hot spot components. KANK1 can bind liprins and LL5β. In turn, liprins bind LL5β and ELKS, while LL5β binds not only ELKS, but CLASP2 on MTs and PIP3 in the membrane, linking the cortical MTs to the membrane and to focal adhesions. Potential association of LL5β with CLASP2 at MT plus ends, with a potential role in kinesin-1-dependent IG delivery, and with CLASP2 at MT lattice, with a potential role in anchoring lateral (looped) MTs for excessive IG withdrawal, is shown. ELKS binds and can alter the current of voltage-dependent Ca2 + channels (VDCCs), which are required for normal vesicle fusion. Figure created with BioRender.com.

Given the known proteins at hot spots, and the regulation of several of them by either the cytoskeleton or the ECM, there is strong evidence for the hot spot to be re-characterized as a “secreting adhesion” (see Figure 1 for model). In short, secreting adhesions consist of scaffolding proteins such as ELKS and liprins (Low et al., 2014; Gan et al., 2017) and key cortical cytoskeleton-associated proteins such as KANK1 and LL5β (Noordstra et al., 2022), among others related to MTs and actin. Importantly, FAK phosphorylation and activation is important for normal clustered secretion from secreting adhesions (Gan et al., 2018; Jevon et al., 2022), but the upstream and downstream pathways are less characterized. This remains true too of the role of the cytoskeleton at secreting adhesions, their role in assembly of secreting adhesions, and their organization and dynamics during clustered secretion. However, as insulin secretion analytical techniques continue to improve, particularly in the field of microscopy, methods such as live imaging of insulin secretion events and super-resolution of the cytoskeleton at secreting adhesions could reveal much of the remaining uncharacterized pathway. Understanding the full pathway by which the β cell accomplishes directed and clustered secretion at secretion adhesions would contribute to our fundamental understanding of glucose homeostasis and therefore elucidate the pathophysiology of diabetes at the secreting adhesion. Its extensive characterization could also identify novel drug targets for therapeutics aiming to improve optimal clustered secretion, a phenomenon which is dysregulated in diabetes (Fu et al., 2019; Cottle et al., 2021).

## Data Availability

The original contributions presented in the study are included in the article/Supplementary Material, further inquiries can be directed to the corresponding author.

## Abbreviations

CAZ, cytomatrix at the active zone; CLASP2, cytoplasmic linker associated protein 2; CMSC, cortical microtubule stabilizing complex; ECM, extracellular matrix; ELKS, rAB6-interacting/CAST family member 1; ERK1/2, extracellular signal-regulated kinase; FAK, focal adhesion kinase; GEF-H1, rho/Rac guanine nucleotide exchange factor 2; GSIS, glucose-stimulated insulin secretion; IG, insulin granule; KANK1, kN motif and ankyrin repeat domains 1; Kif21A, kinesin family member 21A; LAR, leukocyte antigen-related; LL5β, pleckstrin homology like domain family B member 2; MICAL3, microtubule associated monooxygenase, calponin and LIM domain containing 3; MT, microtubule; Myo1b, myosin 1b; PI3K, phosphoinositide 3-kinase; PIP2, phosphatidylinositol 4,5-bisphosphate; PIP3, phosphatidylinositol (3,4,5)-trisphosphate; Rac1, ras-related C3 botulinum toxin substrate 1; RIM2, regulating synaptic membrane exocytosis 2; SNARE, soluble N-ethylmale-imide-sensitive factor-attachment protein receptors; VDCC, voltage-dependent calcium channel.
